# Effect of a Defined Intake of a Soy-Based Protein-Rich Supplement on Body Composition and Eating Behaviour in Overweight Women: A Secondary Analysis of the ACOORH Trial

**DOI:** 10.3390/nu18172809

**Published:** 2026-08-27

**Authors:** Aloys Berg, Andrea Stensitzky-Thielemanns, Hans-Georg Predel

**Affiliations:** 1 Faculty of Medicine, University of Freiburg, 79117 Freiburg, Germany; 2 Independent Researcher, 65307 Bad Schwalbach, Germany; ernaehrung@andrea-stensitzky.de; 3 Institute of Cardiovascular Research and Sports Medicine, German Sport University Cologne, 50933 Cologne, Germany

**Keywords:** overweight, dietary protein intake, meal replacement, body composition, middle-aged women, ACOORH

## Abstract

**Background:** High-protein dietary strategies have been shown to support weight reduction and improve body composition during lifestyle interventions. However, evidence regarding long-term dietary adaptations and their association with body composition in overweight women remains limited. This secondary analysis of the ACOORH randomised controlled trial investigated whether a protein-rich soy-based meal replacement promotes sustained dietary protein intake and favourable body composition changes in female participants. **Methods:** Female participants from the multicentre ACOORH RCT with complete dietary records at baseline, week 12 and week 52 were included (n = 64). Participants received either a standardised lifestyle intervention alone or the same intervention combined with a protein-rich soy–yoghurt–honey meal replacement during the first 26 weeks. Dietary intake, body composition, physical activity and muscle strength were evaluated longitudinally. Between-group comparisons, repeated measures ANCOVA and exploratory correlation analyses were performed. **Results:** Both groups significantly reduced daily energy intake during the first 12 weeks. However, only the intervention group increased body weight-adjusted protein intake (+0.22 ± 0.29 g/kg/day), resulting in a significant between-group difference (*p* = 0.004). The higher protein intake was maintained throughout the 52-week study despite discontinuation of meal replacement after week 26 (*p* < 0.001). Repeated measures ANCOVA demonstrated significantly greater improvements in body mass index (*p* = 0.04) and fat mass (*p* = 0.03) in the intervention group. At week 52, higher protein intake was significantly associated with lower body mass index (r = −0.253; *p* = 0.04), lower fat mass (r = −0.321; *p* = 0.01) and greater muscle strength (r = 0.292; *p* = 0.02), whereas no association with physical activity was observed. **Conclusions:** Among overweight women participating in a structured lifestyle intervention, temporary supplementation with a protein-rich soy-based meal replacement was associated with sustained increases in dietary protein intake and more favourable long-term body composition. These findings support the concept that maintaining a higher protein intake during weight reduction may contribute to improved body composition outcomes. Larger prospective studies specifically designed to investigate sex-specific effects of protein-rich dietary interventions are warranted.

## 1. Introduction

Lifestyle interventions aimed at changing physical activity patterns and promoting a targeted healthy diet have been shown to have clinically significant effects on body composition and metabolic risk factors [1,2]. However, long-term adherence to these measures remains low overall and requires a level of discipline that is unsustainable for most of those affected [3,4]. In this context, the use of meal replacement therapies [5,6,7] has proven to be effective and suitable for patients with obesity and associated comorbidities. As defined high-protein formula diets have proven effective for weight loss, it stands to reason that, in patients with overweight or obesity and associated cardiovascular risk factors, the combination of a high-protein diet as part of a lifestyle intervention is superior to a conventional lifestyle intervention alone in terms of weight loss and the improvement of cardiovascular risk factors [8]. This fact may be of significant interest for overweight middle-aged women who intend to lose weight particularly by reducing their daily total energy intake [9,10] and are therefore at risk of becoming deficient in certain nutrients, in this specific case in their daily protein intake. In addition, sex-specific differences in body composition and protein metabolism may influence the physiological response to dietary interventions. From this perspective, it is of interest to investigate, for an intervention study such as the ACOORH trial [11], the framework of a secondary analysis, possible interactions between eating behaviour and weight control in relation to body composition, physical activity and muscle strength. We hypothesised that female participants receiving the protein-rich soy-based meal replacement would achieve and maintain a higher daily protein intake than participants receiving lifestyle intervention alone and that this sustained dietary adaptation would be associated with a more favourable body composition over the 52-week study period. Accordingly, it was the aim of the present study to prove if a protein-rich diet, achieved by a protein-rich soy-based supplement, may help to stabilise eating behaviour and to improve body composition as well as daily physical activities.

## 2. Methods

### 2.1. Study Design

This secondary analysis was based on female participants of the multicentre randomised controlled ACOORH trial (Almased Concept against Overweight and Obesity and Related Health Risks). The ACOORH study was conducted as an international, multicentre, and randomised controlled trial [11]. The study comprised a 26-week intervention phase and a further 26-week follow-up phase, concluding in week 52. Participants were assigned either to a lifestyle intervention alone (control group) or to a meal-replacement-based lifestyle intervention including a soy–yoghurt–honey protein supplement for 26 weeks. Analyses were restricted to women with complete evaluable dietary records at baseline, week 12, and week 52 (n = 64). The multicentre RCT received ethical approval for each participating centre (registered at drks.de; ID: DRKS00006811). Written informed consent was obtained from all participants. Study participants were recruited at all study centres either through direct contact based on existing patient records or through proactive study enquiries from participants via the study centres’ websites and newspaper advertisements. The inclusion and exclusion criteria were described in detail [11].

### 2.2. Intervention Regimen

**Figure 1:**
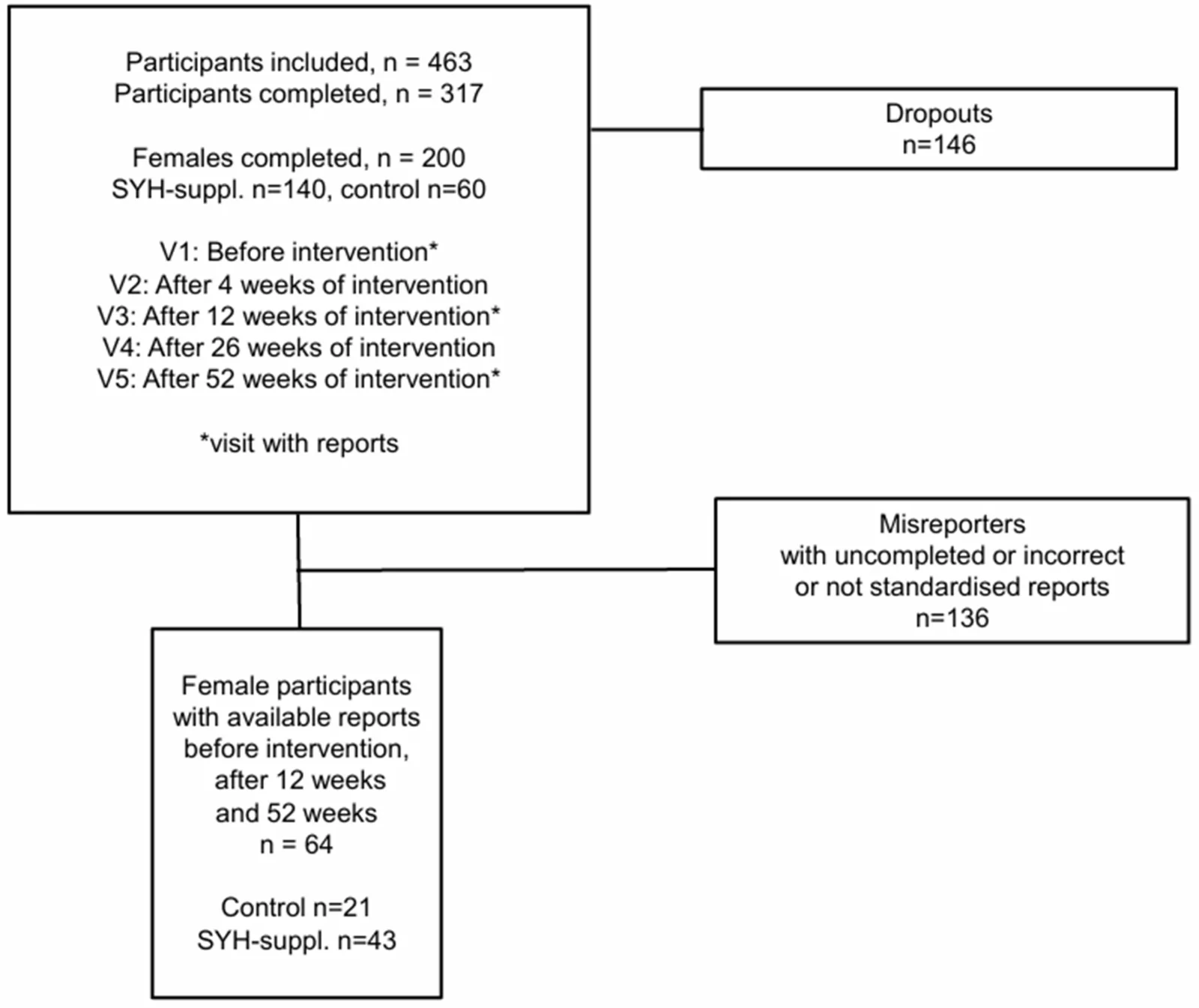
Flow chart.

Both groups were given leaflets containing information on healthy cooking and received advice on physical activity and a healthy lifestyle; all participants were provided with smart scales (smartLAB scale W; HMM Holding AG, Dossenheim, Germany) and pedometers (smartLAB walk P+; HMM Holding AG, Dossenheim, Germany). The participants were also advised to keep a 4-day unweighted food diary [12] at the start of the study and after 12 and 52 weeks, and to record details regarding quality of life [13] and weekly physical activity in a provided log [14]. All records were discussed in detail during the study visits. In addition, the SYH supplement group received a meal replacement (Almased-Vitalkost^®^, Almased Wellness GmbH, München, Germany, a soya–yoghurt–honey-based product: protein content 53.3%) for the first 26 weeks. The manufacture and ingredients of the supplement have already been described in detail elsewhere [7]. Regardless of their group allocation, all participants received regular advice on low-carbohydrate, low-glycaemic and high-protein meals. Evaluable dietary diaries were ultimately available for 64 female participants (Figure 1) across the three visits (before the intervention, after 12 weeks and at the end of the intervention after 52 weeks).

### 2.3. Measurements

All measurements were taken before starting intervention and at 4, 12, 26 and 52 weeks, as described in detail [11]. Body composition (Seca medical Body Composition Analyser^®^ (seca-mBCA 115), Hamburg, Germany and blood pressure (Mobil-O-Graph PWA; I.E.M. GmbH, Stolberg, Germany) were measured using validated devices. Biochemical blood parameters were determined by venous blood sampling in a certified clinical laboratory. Adverse events were continuously documented in a standardised participant questionnaire and reviewed by an external monitor. The primary endpoint of the ACOORH study was body weight in kg after 4, 12, 26 and 52 weeks of intervention. The power calculation was performed for the difference in body weight change after 12 and 52 weeks of intervention between the INT and CON groups. The secondary endpoints were changes in anthropometric parameters and clinical parameters as well as recorded data on dietary habits, physical activity and quality of life before and 12 as well as 52 weeks after intervention [11].

A certified institute (ACOMED statistik^®^, Leipzig, Germany) carried out the statistical analysis; a detailed description, including the statistical tests used (for parametric and non-parametric data) and the software employed, is provided already in [11]. All statistical tests were two-tailed, and significance was set at *α* < 0.05. To assess longitudinal intervention effects throughout the complete 52-week observation period, repeated measures analysis of covariance (ANCOVA) was performed for protein intake and body composition variables. Finally, exploratory Pearson correlation analyses were conducted to examine associations between body weight-adjusted protein intake (g/kg/day) at week 52 and BMI, fat mass, muscle strength, and physical activity.

## 3. Results

Results were calculated for the female participants with available reports before intervention as well as after 12 and 52 weeks. Table 1 shows the descriptive statistics by group and visit for each variable of the performed secondary analyses; Table 2 shows the significance statistics for changes over time and between groups. Both groups showed significant decreases in their daily energy and carbohydrate intake of about 450 kcal/d and 50g/d after the first 12 weeks of intervention. However, a significant difference in protein intake (g/kg/d) was calculated between the groups in this period (*p* = 0.004); in contrast to a decrease at −0.2 g/kg/d in the control group, the protein intake increased at +0.2 g/kg/d in the supplemented group. Significant differences in nutrient intake between the groups were also calculated for the intake (g/d) of glucose, fructose, monosaccharose and saccharose, with a benefit in the supplemented group. These differences in the nutritional behaviour between the groups were combined with significant benefits in the body composition status (*p* < 0.001) during the first 12 weeks in the supplemented group also. In addition, both groups increased their daily physical activity (h/w) significantly within the groups.

**Figure 2:**
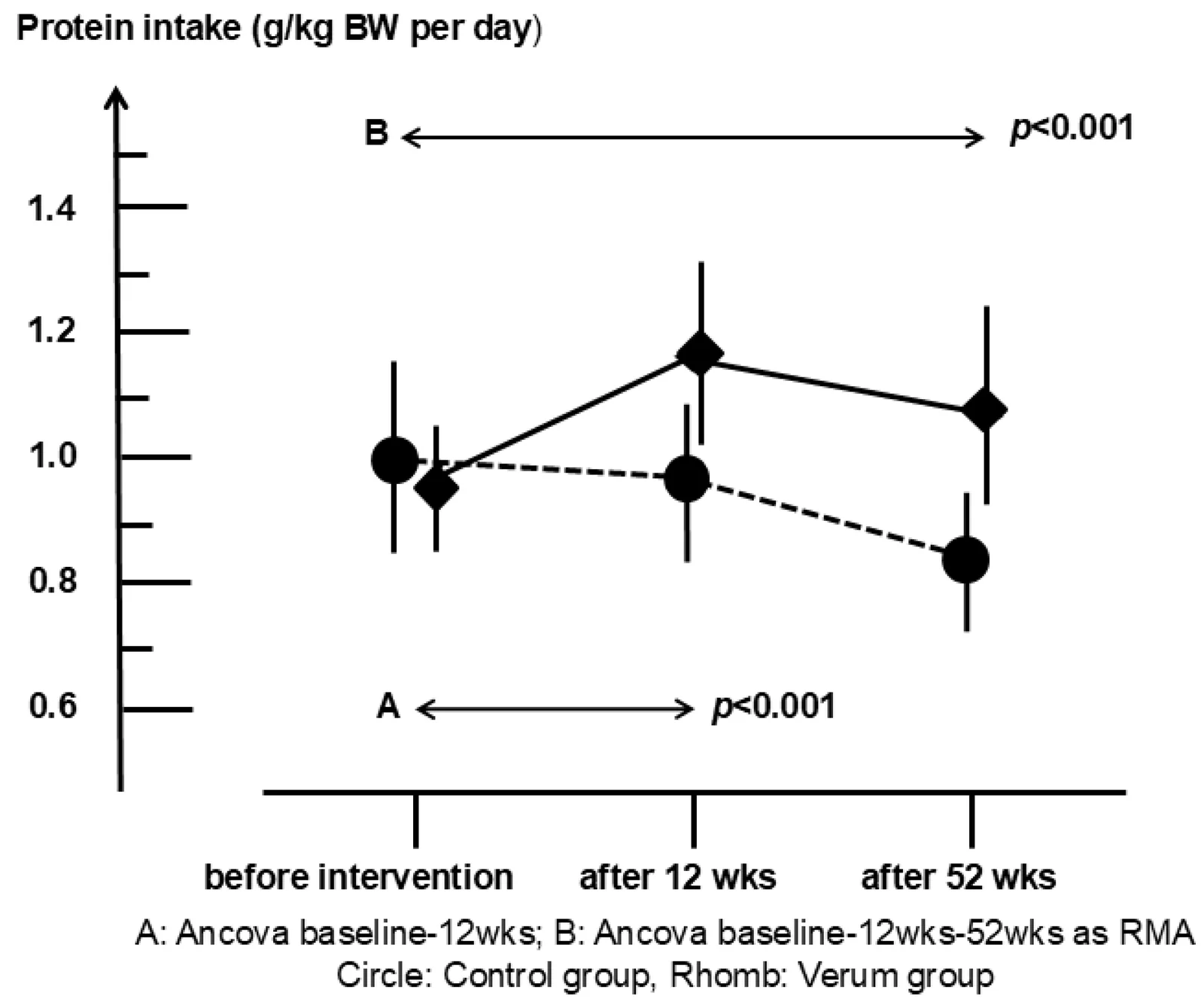
Changes over time between groups after 12 and 52 weeks.

When the significances for changes over time and between groups were calculated for the differences within the 52-week period, significant differences in nutrient intake were only found for total and body weight-related protein intake; these significant differences between the groups (*p* < 0.001; Figure 2) can also be confirmed over the entire study period using ANCOVA with repeated measurements.

It may be of interest that significant differences between groups can also be confirmed over the entire study period by ANCOVA for repeated measurements for body composition parameters, calculated for body mass (control: −1.1 ± 1.66 kg vs. suppl. group: −1.5 ± 1.84 kg; *p* = 0.04) and fat mass also (control: −2.1 ± 3.40 kg vs. suppl. group: −3.2 ± 3.76 kg; *p* = 0.03).

Finally, to explore the relationship between dietary protein intake and body composition, exploratory correlation analyses were performed using body weight-adjusted protein intake at week 52. Higher protein intake was significantly associated with:

Lower body mass index (r = −0.253; *p* = 0.04);
Lower fat mass (r = −0.321; *p* = 0.01);
Greater muscle strength (r = 0.292; *p* = 0.02).

No statistically significant association was observed between protein intake and physical activity.

Taken together, the results demonstrate that temporary supplementation with a protein-rich soy-based meal replacement was associated with sustained increases in dietary protein intake that persisted beyond the active supplementation period. These long-term dietary adaptations were accompanied by more favourable body composition outcomes, particularly lower fat mass, whereas no relationship between protein intake and physical activity was observed.

## 4. Discussion

The present secondary analysis of the ACOORH randomised controlled trial investigated the association between dietary protein intake and long-term body composition in overweight women participating in a structured lifestyle intervention. Three principal findings emerge from the present analyses. First, women receiving the protein-rich soy-based meal replacement achieved a significantly higher dietary protein intake during the intervention period than participants receiving lifestyle intervention alone. Second, this favourable dietary adaptation persisted beyond the active supplementation period and remained evident throughout the 52-week follow-up. Third, sustained higher protein intake was associated with a more favourable body composition, particularly lower fat mass and greater muscle strength. The findings extend previous reports from the ACOORH trial [11]. While the primary analyses demonstrated superior weight reduction, improvements in quality of life, and favourable effects on glucose metabolism, the present secondary analysis provides novel evidence that the intervention was associated with sustained dietary protein intake and more favourable long-term body composition outcomes in women. To our knowledge, few studies have specifically evaluated whether the temporary use of a protein-rich meal replacement induces lasting changes in protein consumption after discontinuation of supplementation.

It can be assumed that, when following a weight loss programme, women tend to respond better to general calorie restriction than to a targeted change in diet involving the restriction of high-fat foods and sweets [9,10]. It can therefore be assumed that the calorie restriction is associated with a reduced protein intake. This restriction in energy intake, particularly when combined with an increased energy output by physical activity, can have negative effects on body composition and quality of life. Ideally, weight loss should primarily come from adipose tissue. However, studies on weight loss programmes have shown that this is usually accompanied by a loss of skeletal muscle mass. Certain groups, including postmenopausal women, older adults, people with metabolic disorders and athletes, may be particularly at risk of losing skeletal muscle mass when participating in a weight management programme. For these groups, a protein intake exceeding the current guideline of 0.8 g/kg body weight/day is therefore recommended. An increase in daily protein intake >1.0–1.3 g/kg/d [8,15] appears to promote fat loss and preserve lean body mass and skeletal muscle mass; such an increase in daily protein intake appears to preserve lean body mass and skeletal muscle mass during weight management programmes. Following our own results of further clinical studies [16], it must be confirmed that the use of the soy–yoghurt–honey formula supports weight loss by the reduction of fat mass while preventing or minimising the simultaneous loss of muscle mass [17].

In the context of a high-protein diet, several physiological mechanisms have to be proposed to explain the beneficial effects of higher dietary protein intake during weight reduction. Particularly, the health benefits of the biologically active peptides (BAPs) supplied by proteins have to be mentioned; these peptides generate metabolic effects at microgram quantities [18,19,20,21]. The soy–yoghurt–honey supplement investigated in these analyses has been shown to contain more than 80 listed bioactive peptides [22] with potential anti-obesity properties. Soy-based foods are known not only as an excellent source of protein but also as a source of biologically active molecules such as phytoestrogens with specific health benefits based on their molecular activities that play a role in body weight regulation [23,24]. There are also study results available for honey, the third ingredient in the supplement used in the intervention examined here, which show that honey [25,26,27] promotes the oxidation of the body’s own fats via the beta-oxidation of muscular fatty acids.

In a similar way to the increased oxidation of muscular fatty acids, own results as well as data from University of Alberta (Edmonton, CA, USA) suggest that consuming the soy–yoghurt–honey product has a positive effect on both appetite regulation as well as hunger scores independent of gender [28,29].

This study has several limitations. First, this was an exploratory secondary analysis and the number of female participants with complete evaluable dietary records across the defined study visits was limited. The reduced sample size increases the risk of selection bias and limits generalisability of the findings. Second, dietary intake was assessed using self-reported food diaries, which are susceptible to reporting inaccuracies and underreporting. Third, multiple statistical comparisons were performed, and the analyses should therefore be considered hypothesis-generating and need to be confirmed by a confirmatory trial. Finally, the correlation analyses describe associations and do not establish causal relationships between dietary protein intake and body composition.

This study also has important strengths. The analyses are based on data from a well-characterised multicentre randomised controlled trial with a 52-week observation period, standardised assessment of dietary intake and body composition, and repeated measurements that enabled evaluation of long-term dietary adaptations. Moreover, restricting the present analysis to women reduced biological heterogeneity and allowed examination of an understudied but clinically relevant subgroup.

Taken together, the present findings support the concept that the maintenance of higher dietary protein intake during structured lifestyle intervention is associated with more favourable long-term body composition outcomes in overweight middle-aged women. Future randomised controlled trials specifically designed to investigate sex-specific responses to higher protein intake should further clarify the optimal quantity, duration, and dietary sources of protein required for sustainable body composition improvement.

## 5. Conclusions

This secondary analysis of the ACOORH randomised controlled trial demonstrates that temporary supplementation with a protein-rich soy-based meal replacement within a structured lifestyle intervention was associated with sustained increases in dietary protein intake among overweight women. Notably, the higher protein intake persisted beyond the supplementation period, indicating that the intervention may have facilitated lasting dietary adaptation.

Women maintaining higher protein intake exhibited more favourable body composition outcomes, including lower fat mass, and dietary protein intake was significantly associated with lower BMI, lower fat mass, and greater muscle strength at end of the study. These findings support the concept that maintaining an adequate protein intake during weight reduction may contribute to improved long-term body composition.

Because this was an exploratory secondary analysis with a relatively small sample size, the observed associations should not be interpreted as causal. Larger prospective randomised studies specifically designed to investigate sex-specific effects of higher dietary protein intake on body composition are warranted to confirm these findings and to define optimal protein recommendations for women undergoing weight management programs.

## Figures and Tables

**Table 1:** Descriptive statistics by group and visit for each variable.

Participants with an Available Nutritional and Lifestyle Protocol at Visit-1, Visit-3 and Visit-5		Control (n = 21)			SYH-Supplement (n = 43)		
		Visit-1	Visit-3	Visit-5	Visit-1	Visit-3	Visit-5
Energy [kcal]	Mean	2123.44	1773.75	1632.05	1940.81	1552.44	1714.09
	SD	647.78	504.70	528.59	581.20	476.31	550.14
Carbohydrates [g]	Mean	223.39	174.54	163.71	189.86	135.44	158.07
	SD	62.93	66.65	62.49	65.06	50.76	62.09
Fat [g]	Mean	88.89	76.58	69.32	85.46	63.77	71.71
	SD	39.65	28.91	26.66	31.67	25.90	26.04
Protein [g]	Mean	87.05	82.21	70.67	80.92	91.87	86.64
	SD	28.02	23.55	19.83	17.89	23.83	26.90
Protein/Body Weight [g/kg]	Mean	0.99	0.97	0.84	0.96	1.17	1.08
	SD	0.29	0.26	0.24	0.22	0.30	0.34
Glucose [g]	Mean	11.24	10.98	10.07	10.50	7.37	9.20
	SD	6.36	4.55	5.53	6.37	4.66	6.58
Fructose [g]	Mean	14.30	14.63	13.04	12.65	9.48	11.47
	SD	8.49	7.58	7.30	8.71	5.76	9.09
Monosaccharide [g]	Mean	26.05	25.99	23.41	23.70	17.40	21.29
	SD	15.00	11.91	12.54	14.56	10.48	15.53
Saccharose [g]	Mean	41.57	32.93	28.83	34.99	21.99	24.31
	SD	19.07	17.41	17.79	20.37	17.20	16.54
Dietary Fibre [g]	Mean	19.21	18.22	16.38	16.65	14.90	16.69
	SD	6.13	6.49	5.93	5.86	6.01	7.72
Body Weight [kg]	Mean	87.90	84.71	84.71	84.97	78.61	80.84
	SD	10.13	9.59	11.39	7.75	8.20	9.89
BMI [kg/m^2^]	Mean	31.10	29.98	29.97	31.15	28.81	29.62
	SD	2.40	2.37	3.12	2.66	2.88	3.42
Fat Mass [kg]	Mean	39.06	36.48	36.96	37.41	32.31	34.24
	SD	6.06	5.88	7.09	6.28	6.70	7.83
Lean Body Mass [kg]	Mean	48.83	48.23	47.74	47.53	46.29	46.58
	SD	5.53	5.70	5.74	4.05	3.63	4.01
Skeletal Muscle Mass [kg]	Mean	23.75	23.29	23.08	22.96	21.87	22.19
	SD	3.28	3.28	3.40	2.43	2.33	2.51
Strength [kg]	Mean	26.41	26.59	26.55	26.56	27.46	27.66
	SD	7.10	6.89	7.66	6.19	6.46	6.69
Physical Activity [hours/week]	Mean	5.96	14.64	6.27	7.41	10.47	11.24
	SD	4.88	14.50	3.70	7.55	7.67	11.53

**Table 2:** Significance statistics for changes over time and between groups (n.sign.: not significant).

Participants with Nutritional and Lifestyle Data Control n = 21 Suppl. Group n = 43	Differences over Time and Significant Changes Within Groups				Significant Diff. Between Groups Adjusted for Baseline Value (ANCOVA/Repeated Measurement)		
	Diff. V3-V1		Diff. V5-V1		Diff.V3-V1	Diff.V5-V1	Rep. Measurm.
	Control [M/SD]	Suppl. [M/SD]	Control [M/SD]	Suppl. [M/SD]	Suppl.-Control	Suppl.-Control	Suppl.-Control
Energy [kcal]	−349/639 *p* = 0.017	−388/601 *p* < 0.001	−491/390 *p* < 0.001	−226/610 *p* = 0.022	n.sign.	n.sign.	n.sign.
Carbohydrate [g]	−48/60.1 *p* < 0.001	−54/66.8 *p* < 0.001	−60/45.3 *p* < 0.001	−32/71.9 *p* = 0.004	n.sign.	n.sign.	n.sign.
Fat [g]	−12/41.6 n.sign.	−22/31.2 *p* < 0.001	−20/25.9 *p* = 0.003	−14/29.6 *p* = 0.005	n.sign.	n.sign.	n.sign.
Protein [g]	−4.8/29.7 n.sign.	10.9/23.9 *p* = 0.006	−16/21.6 *p* = 0.001	5.7/26.2 n.sign.	n.sign.	*p* = 0.002	*p* = 0.002
Prot./BW [g/kg]	−0.2/0.32 n.sign.	0.22/0.29 *p* < 0.001	−0.2/0.24 *p* = 0.009	0.12/0.32 *p* = 0.011	*p* = 0.004	*p* < 0.001	*p* < 0.001
Glucose [g]	−0.3/6.33 n.sign.	−3.1/5.97 *p* < 0.001	−1.2/5.65 n.sing.	−1.3/7.39 n.sign.	*p* = 0.004	n.sign.	n.sign.
Fructose [g]	0.32/9.36 n.sign.	−3.2/8.76 *p* 0 0.011	−1.3/8.58 n.sign.	−1.2/10.8 n.sign.	*p* = 0.005	n.sign.	n.sign.
Monosacch. [g]	−0.1/15.6 n.sign.	−6.3/14.6 *p* < 0.004	−2.6/14.0 n.sign.	−2.4/17.9 n.sign.	*p* = 0.005	n.sign.	n.sign.
Saccharose [g]	−8.6/19.4 n.sign.	−13/25.3 *p* = 0.001	−12/13.0 *p* < 0.001	−11/23.9 *p* = 0.005	*p* = 0.037	n.sign.	n.sign.
Diet. Fibre [g]	−1.0/6.59 n.sign.	−1.8/4.75 *p* = 0.024	−2.8/5.91 *p* = 0.042	0.03/7.61 n.sign.	n.sign.	n.sign.	n.sign.
Body Weight [kg]	−3.2/3.37 *p* < 0.001	−6.4/3.60 *p* < 0.001	−3.2/4.63 *p* = 0.006	−4.1/4.70 *p* < 0.001	*p* < 0.001	n.sign.	n.sign.
BMI [kg/m^2^]	−1.1/1.20 *p* < 0.001	−2.3/1.37 *p* < 0.001	−1.1/1.66 *p* = 0.010	−1.5/1.84 *p* < 0.001	*p* < 0.001	n.sign.	*p* = 0.040
Fat Mass [kg]	−2.6/2.77 *p* < 0.001	−5.1/2.77 *p* < 0.001	−2.1/3.40 *p* = 0.009	−3.2/3.76 *p* < 0.001	*p* < 0.001	n.sign.	*p* = 0.028
LB-Mass [kg]	−0.6/1.56 n.sign.	−1.2/1.34 *p* < 0.001	−1.1/1.60 *p* = 0.006	−0.9/1.35 *p* < 0.001	*p* = 0.047	n.sign.	n.sign.
Skelet.MM [kg]	−0.5/1.08 n.sign.	−1.1/1.14 *p* < 0.001	−0.7/1.23 *p* = 0.021	−0.7/1.39 *p* < 0.001	*p* = 0.018	n.sign.	n.sign.
Strength [kg]	0.18/3.21 n.sign.	0.71/4.04 n.sign.	0.14/3.18 n.sign.	1.10/3.67 n.sign.	n.sign.	n.sign.	n.sign.
Phys. Act. [h/w]	8.7/13.2 *p* = 0.004	3.4/6.43 *p* = 0.001	0.16/3.44 n.sign.	3.93/11.4 n.sign.	n.sign.	n.sign.	n.sign.

## Data Availability

The datasets generated during and/or analysed during the current study are not publicly available but are available from the corresponding author on reasonable request.
